# Evidence for Altered Ca^2+^ Handling in Growth Associated Protein 43-Knockout Skeletal Muscle

**DOI:** 10.3389/fphys.2016.00493

**Published:** 2016-10-26

**Authors:** Giusy A. Caprara, Caterina Morabito, Stefano Perni, Riccardo Navarra, Simone Guarnieri, Maria A. Mariggiò

**Affiliations:** ^1^Laboratory of Functional Biotechnology, Center of Sciences on Aging and Translational Medicine (CeSI-MeT), Department of Neuroscience, Imaging and Clinical Sciences, University G. d'Annunzio of Chieti-PescaraChieti, Italy; ^2^Department of Cell and Developmental Biology, Perelman School of Medicine, University of PennsylvaniaPhiladelphia, PA, USA; ^3^Department of Neuroscience, Imaging and Clinical Sciences, University G. d'Annunzio of Chieti-PescaraChieti, Italy

**Keywords:** skeletal muscle, GAP43, calmodulin, intracellular calcium, muscle differentiation

## Abstract

Neuronal growth-associated protein 43 (GAP43) has crucial roles in the nervous system, and during development, regeneration after injury, and learning and memory. GAP43 is expressed in mouse skeletal muscle fibers and satellite cells, with suggested its involvement in intracellular Ca^2+^ handling. However, the physiological role of GAP43 in muscle remains unknown. Using a GAP43-knockout (GAP43^−/−^) mouse, we have defined the role of GAP43 in skeletal muscle. GAP43^−/−^ mice showed low survival beyond weaning, reduced adult body weight, decreased muscle strength, and changed myofiber ultrastructure, with no significant differences in the expression of markers of satellite cell and myotube progression through the myogenic program. Thus, GAP43 expression is involved in timing of muscle maturation *in-vivo*. Intracellular Ca^2+^ measurements *in-vitro* in myotubes revealed GAP43 involvement in Ca^2+^ handling. In the absence of GAP43 expression, the spontaneous Ca^2+^ variations had greater amplitudes and higher frequency. In GAP43^−^/^−^ myotubes, also the intracellular Ca^2+^ variations induced by the activation of dihydropyridine and ryanodine Ca^2+^ channels, resulted modified. These evidences suggested dysregulation of Ca^2+^ homeostasis. The emerging hypothesis indicates that GAP43 interacts with calmodulin to indirectly modulate the activities of dihydropyridine and ryanodine Ca^2+^ channels. This thus influences intracellular Ca^2+^ dynamics and its related intracellular patterns, from functional excitation-contraction coupling, to cell metabolism, and gene expression.

## Introduction

Neuronal growth-associated protein 43 (GAP43) was originally considered to be a neuron-specific marker. It has been described as having crucial roles in the nervous system, and during development, in regeneration after injury, and in learning and memory processes (Mosevitsky, 2005).

In different species, the GAP43 protein has 194–238 amino-acids, the sequence of which is characterized by three functional domains: (i) the C-terminal domain, which is known as neuromodulin and is specific to GAP43; (ii) the 57-amino-acid N-terminal region, that is highly conserved in all vertebrates, and where the first 10 amino acids (the membrane-binding region) are crucial for its binding to the nerve-terminal membrane (Zuber et al., 1989); and (iii) the domain, following the N-terminal region, that is known as the IQ domain, of 12–15 amino acids, and which binds calmodulin (CaM), a Ca^2+^-binding protein involved in cellular processes that are modulated by intracellular Ca^2+^ (Oestreicher et al., 1984; Jacobson et al., 1986; Alexander et al., 1987; Benowitz et al., 1987; Coggins and Zwiers, 1989; Zuber et al., 1989; Chapman et al., 1991).

The IQ domain of GAP43 starts at the isoleucine and glycine that lie just prior to its Ser41 and it extends over about 12–15 amino acids toward the C-terminus (Oestreicher et al., 1984; Jacobson et al., 1986; Alexander et al., 1987; Benowitz et al., 1987; Coggins and Zwiers, 1989; Zuber et al., 1989; Chapman et al., 1991). This Ser41 is crucial, because it is the only site that can be phosphorylated by protein kinase C (PKC) (Coggins and Zwiers, 1989; Chapman et al., 1991). Due to its sequence, GAP43 appears to be the union of two different proteins: a palmitoylation substrate protein, and a phosphorylation substrate protein (Denny, 2006). It has been proposed that GAP43 serves as a CaM “sponge,” through sequestration of CaM to sites near the nerve-terminal membrane, and its release after GAP43 phosphorylation, in response to second messengers. If PKC is not sufficiently activated by transient changes in second messengers (e.g., Ca^2+^, diacylglycerol, arachidonic acid), the Ser41 is not phosphorylated, and CaM remains linked to GAP43 (Sheu et al., 1995). This might then serve to prevent GAP43 from being activated in response to transient changes in second messengers. Conversely, the Ser41 phosphorylation modulates intracellular CaM levels and GAP43 supplies a scaffold to localize CaM near to the membrane domains. However, once PKC becomes sufficiently activated to phosphorylate Ser41, CaM is released from GAP43. This maintains GAP43 in the activated state even if the second messenger levels decline. Once released from GAP43, CaM becomes available to stimulate the substrate proteins of CaM kinase in nerve terminals (provided the intracellular Ca^2+^ is sufficiently high). In a feedback loop, the activation of one target protein, calcineurin, results in GAP43 de-phosphorylation and CaM re-association (Benowitz and Routtenberg, 1997).

GAP43 has also been reported for non-nervous tissues, and in particular in embryonic chicken limb and in human skeletal muscle biopsies (Stocker et al., 1992a,b; Heuss et al., 1994, 1995; Heuss and Schlötzer-Schrehardt, 1998). Recently, we showed that GAP43 is expressed in both skeletal muscle cell lines and satellite cells, as well as in isolated mouse muscle fibers (Guarnieri et al., 2013). Data obtained from skeletal muscle cell models have suggested that during the differentiation process, the distribution of GAP43 changes from predominantly nuclear localization to the cytoplasmic compartment, in the form of double striations. GAP43 is also seen in regular double striations in fibers of the mouse *extensor digitorum longus* (EDL) muscle, and which runs parallel with the mitochondria and Ca^2+^-release units (CRUs) (Guarnieri et al., 2013). Of note, these CRUs are macromolecular complexes that are located in the triads, which are structures formed by the juxtaposition of the T-tubule and a pair of terminal cisternae of the sarcoplasmic reticulum. These contain the dihydropyridine receptor Ca^2+^ channel (DHPR) and the ryanodine receptor (RyR), which are essential for the excitation-contraction processes (Franzini-Armstrong and Jorgensen, 1994).

Further investigations into the expression and localization of GAP43 in skeletal muscle of amphibians and fish have clarified the positioning of GAP43 relative to these CRUs and the mitochondria. Amphibians and fish are known to have triads at the level of the Z-lines, and with which the mitochondria are not closely associated (Franzini-Armstrong and Porter, 1964; Franzini-Armstrong, 1973; Franzini-Armstrong et al., 1999). In skeletal muscle of Zebrafish and in *Xenopus*, GAP43 is localized in single transverse and fairly narrow bands at the Z-line, which reflects the disposition of the CRUs in these models, and confirms the direct correlation between GAP43 and the CRUs (Franzini-Armstrong and Porter, 1964; Franzini-Armstrong, 1973; Franzini-Armstrong et al., 1999; Caprara et al., 2014).

To date, the physiological role of GAP43 in muscle has remained unknown. Its particular localization in skeletal muscle indirectly suggests its involvement in Ca^2+^/calmodulin homeostasis, particularly with the strong evidence of GAP43 and CaM interactions in the nervous system.

In the present study, a GAP43-knockout (KO) mouse model and primary myotube cultures were used and the data demonstrate that GAP43 is necessary for the normal timing of the physiological development of skeletal muscle, and that GAP43 has a key role in intracellular Ca^2+^ homeostasis.

## Materials and methods

### Chemicals and materials

Unless otherwise indicated, cell-culture media, sera, and antibiotics were from EuroClone S.p.A. (Pero, Italy), cell-culture plastic-ware was from Becton Dickinson Falcon (Sacco Srl, Cadorago, Italy), and reagents and standards were from Sigma-Aldrich (Milan, Italy).

### *In-vivo* analyses

#### Animal models

C57BL/6 GAP43 heterozygous (+/−) mice were kindly provided by Karina F. Meiri (State University of New York, USA). The care and use of these mice and the generated wild-type (WT) and homozygous (−/−) mice strictly followed “The Guiding Principles for the Care and Use of Animals,” in accordance with the principles of the Declaration of Helsinki and with the European Community Council (86/609/CEE) and the Italian Government law on the protection of animals for experimental procedures in research laboratories (92/116). All of the procedures were also approved by the local University Committee on Animal Resources, Comitato Etico Interistituzionale per la Sperimentazione Animale (CEISA) (prot. n. 15/2011/CEISA/COM). The mice were housed and tested in the animal facility of the Centre for Research on Aging (Chieti, Italy), and were sacrificed by cervical dislocation.

#### Genotyping

The mouse litters were genotyped using a small fragment of the tails of neonatal or 3-week-old mice, to genotype the offspring of the GAP43-heterozygote crosses. DNA extractions and amplifications were performed using mouse genotyping kits (KAPA Biosystems, Resnova S.r.l., Genzano di Roma, Italy), according to the manufacturer instructions. The DNA samples were amplified by PCR using the following primers: p1 (5′-GGCTCATAAGGC TGCAACCAAAAT-3′); p2 (5′-CCATCTCCCTCC TTCTTCTCCACA-3′); p3 (5′-CCGGCCGCTTGG GTGGAGAG-3′); and p4 (5′-TCGGCAGGAGCA AGGTGAGATGAC-3′).

#### Body weight and grip strength test

All of the mice tested were weighed daily before the grip strength test. Grip strength was evaluated using a force transducer (Shimpo Fgv 0.5 ×; Metrotec Group, San Sebastian, Spain), by lifting the mouse and allowing it to grasp a grid with its paws. Then the mouse was gently pulled by the tail until it let go of the grid. The force of resistance was measured in a single three-trial session, and the strongest measure was recorded as the best run.

### *Ex-vivo* analyses

#### Electron microscopy

The mice were sacrificed and their diaphragms and EDL muscles were dissected out and fixed in 4% glutaraldehyde in 0.1 M sodium cacodylate buffer. After washings with 0.1 M sodium cacodylate buffer at room temperature, the samples were incubated for 1 h at 4⋅C in 2% OsO_4_ in 0.1 M sodium cacodylate buffer, and after water washings, they were incubated for 2 h in saturated aqueous uranyl acetate solution at room temperature. After dehydration through graded ethanol concentrations and acetone, the diaphragms and EDL muscles were embedded in epoxy resin (Epon 812; Polyscience, Niles, USA) for longitudinal and cross sectioning. The sections were cut with a microtome (Ultracut R; Leica Microsystem, Vienna, Austria) using a diamond knife (Diatome Ltd., Biel, Switzerland), and then stained in 4% uranyl acetate before analysis in an electron microscope (Model 410; Philips Electron Optics, Mahwak, USA).

#### Immunofluorescence analysis

The diaphragms and EDL muscles were fixed in 4% paraformaldehyde, permeabilized with 0.2% Triton X-100, incubated in blocking buffer (10% goat serum in phosphate-buffered saline) and stained with the primary antibody (rabbit polyclonal anti-synaptophysin; 1:500 dilution; Abcam, Cambridge, UK; HPA-GAP43 rabbit polyclonal anti-GAP43; 1:500 dilution; Sigma-Aldrich; or mouse monoclonal anti-CaM; 1:50 dilution; Merck Millipore, Darmstadt, Germany). The primary antibodies were revealed using the specific secondary antibody (goat anti-rabbit or anti-mouse Alexa Fluor-488, or goat anti-rabbit Alexa Fluor-568; 1:200 dilution, Life Technologies, Monza, Italy). The diaphragms were then incubated with 2 μg/ml α-bungarotoxin tetramethylrhodamine conjugate (Life Technogies), for 30 min at room temperature.

Images were acquired using a Zeiss system (LSM 510 META; Jena, Germany) equipped with an inverted microscope (Zeiss Axiovert 200) and an oil-immersion objective (Plan Neofluar; 40 × /1.3 NA, 100 × /1.3 NA). Quantitative analyses on the acquired images were obtained using the Zeiss LSM software version 3.0. The data for the fluorescence image profiles were obtained using the LSM 3.0 image analysis software (Zeiss).

### *In-vitro* analyses

#### Satellite cell isolation

The mice were sacrificed and the hind-limb muscles were harvested under sterile conditions, and then manually shredded and digested in 0.05% trypsin/0.02% EDTA. The suspension containing the released cells was collected and after filtration through a 40-μm cell strainer (BD Falcon), the cell suspension was centrifuged (200 × g, 10 min). The cell pellet was re-suspended in growth medium [Dulbecco's modified Eagle's medium supplemented with 20% horse serum, 3% chicken-embryo extract (Seralab, Sussex, UK), 100 IU/ml penicillin, 100 μg/ml streptomycin, and 2 mM L-glutamine]. After a 1-h pre-plating, the satellite cells were plated in collagen-coated dishes in growth medium. After 3 days, the growth medium was changed to the differentiation medium (Dulbecco's modified Eagle's medium supplemented with 2% horse serum) to obtain the differentiated myotubes (Guarnieri et al., 2014).

#### Fusion index

The level of differentiation of the satellite cells into myotubes was determined using immuno-staining protocols with an antibody against myosin heavy chain (MF20; mouse anti-MHC; 1:200 dilution; Hybridoma Bank, Iowa, USA) and an antibody against MyoD (rabbit polyclonal antibody; 1:50 dilution; Santa Cruz Biotechnology Inc, Santa Cruz, USA), accompanied by ToPro dye (1:1000 dilution; Life Technologies), for the staining of the nuclei. The number of ToPro-stained nuclei located within the sarcomeric myosin-positive cells, and the MyoD-positive cells, were counted and expressed as the Fusion Index (Guarnieri et al., 2014).

#### Western blotting

Protein extracts for Western blotting were isolated from the brains and limb muscles of the mice, and from their undifferentiated satellite cells and myotubes. The tissues were homogenized (T10 basic Ultra Turrax IKA-Werke GmbH & Co. KG, Staufen, Germany) in ice-cold lysis buffer (50 mM Tris-HCl, 100 mM NaCl, 50 mM NaF, 40 mM β-glycerophosphate, 5 mM EDTA, 1% Triton X-100, 200 μM sodium orthovanadate, 100 μg/ml phenylmethylsulfonyl fluoride, 10 μg/ml leupeptin, 5 μg/ml pepstatin-A, 10 μg/ml benzamidine, pH 7.4), and the cells were lysed in ice-cold lysis buffer with vortexing. After centrifugation (400 × g, for 10 min at 4⋅C), the protein concentrations in the supernatants were determined (Bio-Rad protein assay; Bio-Rad Laboratories S.r.l, Segrate, Italy). Samples (40 μg) were resuspended in Laemmli buffer and separated by SDS-PAGE on 10% (w/v) homogeneous slab gels, and then electroblotted onto nitrocellulose membranes (AmershamTM-HybondTM-ECL; GE Healthcare, Milan, Italy). Equal loading of the protein samples and transfer efficiency were monitored using Red Ponceau S (Sigma-Aldrich) staining of each membrane, and Coomassie blue staining (0.25% Coomassie blue solution; R 250/G 250 1:1; Bio-Rad) of the gels.

The membranes were blocked, and then incubated with the primary antibodies: anti-GAP43 (HPA-GAP43 rabbit polyclonal antibody; 1:1000 dilution, Sigma-Aldrich); anti-myosin heavy chain (MF20 anti-MHC; 1:1000 dilution, Hybridoma Bank); anti-Pax7 (mouse monoclonal; 1:5 dilution; kindly provided by Antonio Musarò); anti-MyoD (rabbit polyclonal antibody; 1:500 dilution; Santa Cruz Biotechnology); or anti-MyoG (F5D mouse monoclonal; 1:200 dilution; Hybridoma Bank). The membranes were then incubated with horseradish-peroxidase-conjugated anti-IgG, with the relevant proteins detected using chemiluminescence kits (Pierce EuroClone S.p.A., Italy) and an image acquisition system (Uvitec, Cambridge, UK). An anti-GAPDH antibody (1:2000 dilution; Millipore, Vimodrone, Milan, Italy) was used as the loading control.

#### Intracellular Ca^2+^ analysis

Intracellular Ca^2+^ levels were monitored in myotubes using the Fluo4-acetoxymethyl ester dye (Fluo4/AM; Life Technologies). An upright microscope (Zeiss Axio Examiner) was used, equipped with a 40 × 0.75NA water-immersion objectives and connected by an optical fiber to a 75W Xenon lamp and a monochromator (OptoScan; Cairn Instrument, UK). The fluo4-loaded cells in normal external solution (140 mM NaCl, 2.8 mM KCl, 2 mM CaCl_2_, 2 mM MgCl_2_, 10 mM glucose, 10 mM Hepes, pH 7.3) were excited at 488 nm, and the fluorescence images were acquired at 5 frames/s with a 16 bit digital EMCCD camera (PhotoEvolve 512; Photometrics; Tucson, AZ, USA).

The temporal analysis was calculated as the mean fluorescence intensity signal in a selected cell area, as f/f_0_, where f is the fluorescence emission of a single loaded cell that was acquired during the time lapse, and f_0_ is the mean fluorescence intensity of the same cell calculated from images acquired during the first 5 s. The time to peak was also recorded, and the velocity to the peak was calculated as the ratio between the peak amplitude and the time to peak.

#### Electrophysiological measurements

In voltage-clamp experiments, Ca^2+^ currents were recorded using an extracellular solution containing 10 mM CaCl_2_, 5 mM glucose, 120 mM tetraethylammonium chloride, 10 mM Hepes, 1 mM MgCl_2_, 0.1 mM ethylene glycol-bis-b-aminoethyl ether-N,N,N',N'-tetraacetic acid (EGTA), pH 7.4. The patch pipette was filled with 130 mM CsCl, 1 mM EGTA, 0.5 mM MgCl_2_, 10 mM Hepes, pH 7.4. During recordings, the bath contained 2 μM tetrodotoxin, to prevent Na^+^ current activation. The electrophysiological recordings were carried out at room temperature using the whole-cell configuration of the patch-clamp technique. Stimulation, acquisition, and data analysis were performed with the pCLAMP 9.0 and Clampfit version 10.0 software (Axon Instruments, Burlingame, USA). The linear components of leak and capacitive currents were canceled using the P/N4 method. The patch pipettes had resistances of 3–6 MΩ. To abolish the T-type contribution and select L-type Ca^2+^ currents, the cells were clamped from the holding potential of −90 to −30 mV for 750 ms, then a ramp was applied from −60 to +60 mV, with 10 mV increments and of 500 ms duration. A sampling interval of 50 kHz (20 μs/point) was used, and the currents were filtered at 2 kHz (Bessel filter).

### Statistical analyses

#### Sample size

Due to the specific features of the GAP43 KO and WT mice, the sample sizes were variable, and depended on the phenotype and age, as well as on the experimental protocols. The samples and sizes are summarized in Table 1.

**Table 1:** **Sample sizes for the experimental systems used in this study**.

**Experimental system**	**Sample size (n)**								
	**WT**			**+/−**			**−/−**		
Body weight	18–68			29–73			2–49		
Grip test	18–68			29–61			2–4		
Electron microscopy	48–202 fibers	241–322 myofibers	124–164 triads	66–191 fibers	149–291 myofibers	107–211 triads	147–281 fibers	229–269 myofibers	143–194 triads
Morphological analyses, immunostaining, western blotting	Muscles or three cell cultures from three newborn mice, for at least three independent experiments								
Intracellular Ca^2+^	71–83 myotubes from at least 6 independent experiments per condition			69–85 myotubes from at least 6 independent experiments per condition			59–65 myotubes from at least 6 independent experiments per condition		
Electrophysiology	21–25 myotubes from at least three independent experiments per condition			17–21 myotubes from at least three independent experiments per condition			19–23 myotubes from at least three independent experiments per condition		

*The body weight and grip test were performed in each phenotype and the number range represents the lowest and highest numbers of animals tested over the first year. For electron microscopy analyses, three newborns per phenotype were sacrificed, and for each sample, the number range represents the lowest and highest numbers of fibers, myofibers, and triads analyzed in different sections. Morphological analyses, immunostaining, and Western blotting were performed in triplicate. Intracellular Ca^2+^ analyses and electrophysiological recordings were performed in at least three or six independent experiments on myotubes deriving from three newborns per phenotype; the number range represents the lowest and highest numbers of myotubes tested. WT, wild type mice; +/− and −/−, GAP43 heterozygous and homozygous mice, respectively*.

#### Statistics

All of the quantitative data were analyzed using the Prism 4 software (GraphPad Software, San Diego, CA, USA). Data are expressed as means ± standard error of the mean (SEM), and were compared using Students' *t*-tests. The data expressed as frequency distributions were compared using Chi-square tests for trends.

## Results

### Effects of GAP43 deletion on body weight and muscle strength in mice

The GAP43^−/−^ mice were born in the expected Mendelian ratio from GAP43^+/−^ mice. As reported in other studies (Strittmatter et al., 1995; Metz and Schwab, 2004), more than 50% of the GAP43^−/−^ mice died within 2 days of birth (P0–P2), and most of the remaining died between P14 and P21, with only about 5% that survived weaning.

**Figure 1:**
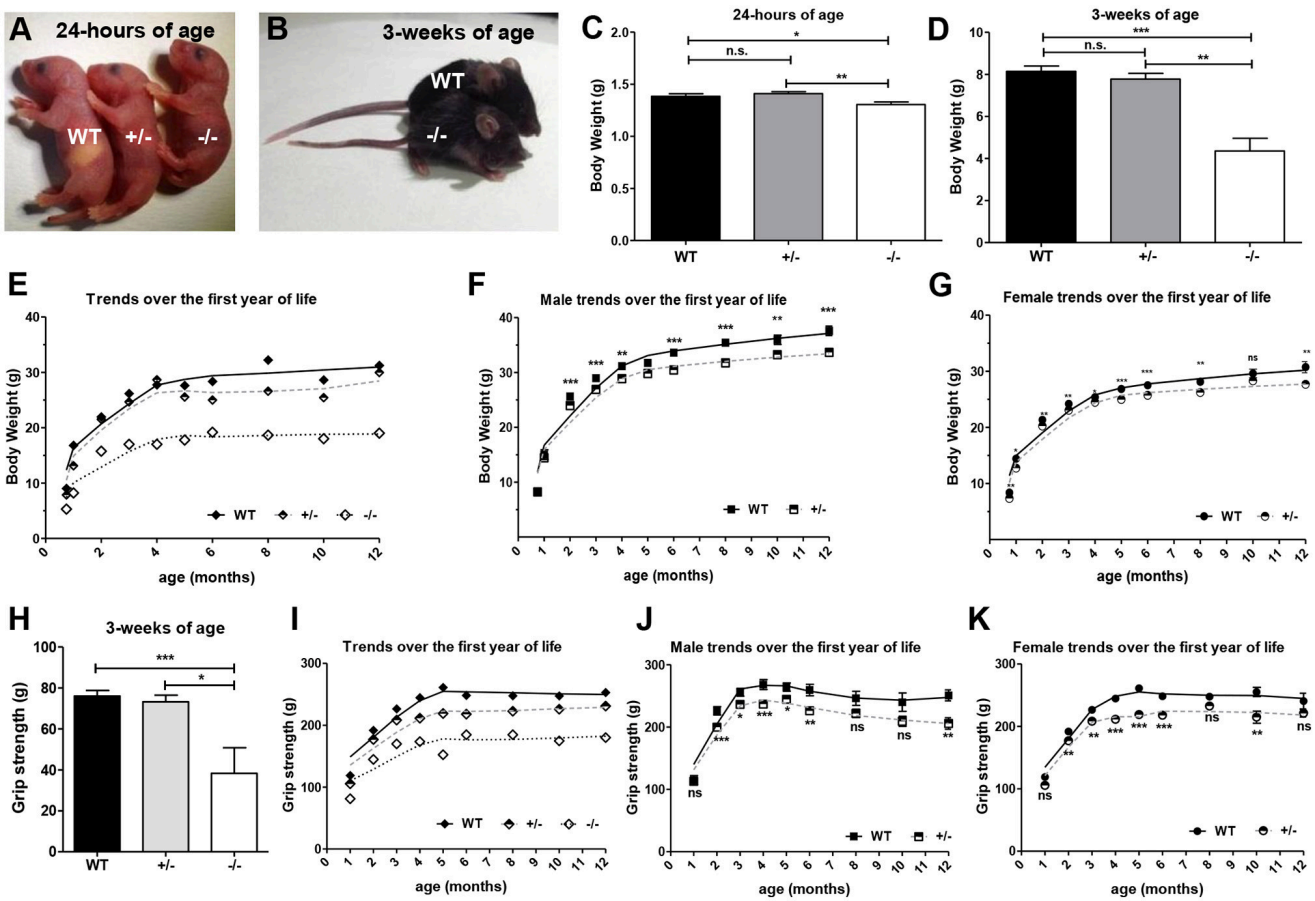
**Body weight and muscle strength of the WT, GAP43^+/−^ (+/−), and GAP43^−/−^ (−/−) mice. (A,B)** Representative wild type mice (WT), and GAP43 heterozygous (+/−) and homozygous (−/−) mice at 24 h **(A)** and 3 weeks of age **(B)**. **(C,D)** Body weight of the mice, as illustrated in **(A,B)**. **(E–G)** Body weight over the first year of life for three representative mice from the same litter **(E)**, and for male **(F)** and female **(G)** WT and GAP43^+/^^−^ mice. **(H)** Absolute muscle strength according to the grip test in 3-week-old mice. **(I–K)** Absolute muscle strength over the first year of life for three representative mice from the same litter **(I)**, and for male **(J)** and female **(K)** WT and GAP43^+/^^−^ mice. Data are means ± SEM **(C,D,F,G,H,J,K)**. ^*^p < 0.05; ^**^p < 0.01; ^***^p < 0.001 (Students' *t*-tests).

At birth, the GAP43^−/−^ mice were not visually distinguishable from their WT or GAP43^+/−^ littermates, although their body weights were slightly lower than the WT and GAP43^+/−^ newborn mice (Figures 1A,C). During the early weeks of life, the differences in the body weights increased (Figures 1B,D), as also in the adult mice (from 3 weeks old, to 1 year-old; Figure 1E). In particular, the GAP43^−/−^ mice did not increase in weight as much as the WT and GAP43^+/−^ mice, even if the feeding appeared normal across all of these phenotype populations. The GAP43^+/−^ mice had lower body weighs in comparison to the WT mice, with significant differences within the genders (Figures 1F,G).

In addition to being smaller, the adult GAP43^−/−^ mice had more pointed faces and a “hunchback” posture. When hung by the tail with the head pointing down, these mice showed different body posture reflexes: WT mice responded by raising the head and extending both fore and hind-limbs; GAP43^−/−^ mice showed reduced reflexive head movements and retracted the hind-limbs into the body.

The grip strength test provides a simple way to determine global muscle performance during maximal isometric contraction of short duration (Connolly et al., 2001). At 3 weeks of age, the GAP43^−/−^ mice performed poorly in the grip test, compared to the GAP43^+/−^ and WT mice (Figure 1H).

These grip tests revealed that also as adults these GAP43 KO mice showed decreased absolute muscle strength (Figure 1I). Likewise, the GAP43^+/−^ mice showed worse performances with respect to WT mice, for both males and females, and from 2 months of age (Figures 1J,K).

### Ultrastructural analysis of skeletal muscle features of the GAP43^−/−^ mice

**Figure 2:**
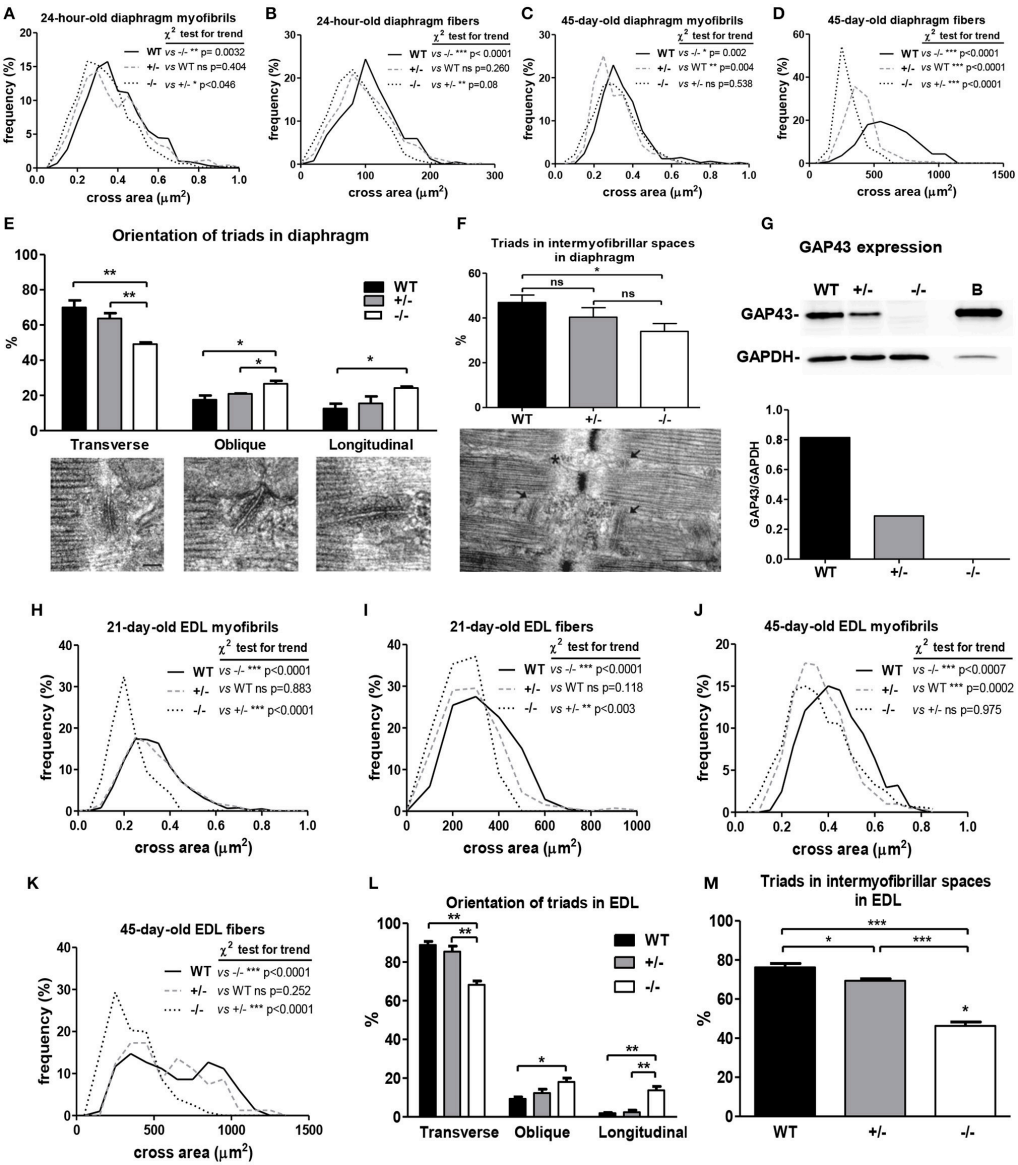
**Morphometric and morphological analyses of the diaphragm and EDL muscles from the WT, GAP43^+/−^ (+/−), and GAP43^−/−^ (−/−) mice. (A–D)** Relative size frequencies of myofibrils **(A,C)** and fibers **(B,D)** in cross-sections of diaphragms from 24-h-old **(A,B)** and 45-day-old **(C,D)** mice. p: Chi-square tests for trends; myofibrils in cross-sections of diaphragms from 24-h-old mice, n: 870 WT, 669 (+/−) and 760 (−/−); fibers in cross-sections of diaphragms from 24-h-old mice, n: 385 WT, 421 (+/−) and 633 (−/−); myofibrils in cross-sections of diaphragms from 45-day-old mice, n: 340 WT, 330 (+/−) and 389 (−/−); fibers in cross-sections of diaphragms from 45-day-old mice, n: 139 WT, 140 (+/−) and 92 (−/−). **(E)** Percentages of transverse, oblique and longitudinal triads (e.g., see bottom representative images) in longitudinal sections of diaphragm fibers from 24-h-old mice. Bar: 100 nm. **(F)** Percentages of triads localized in the intermyofibrillar space (e.g., see arrows in bottom representative image) in longitudinal sections of diaphragm fibers from 24-h-old mice. Bar: 100 nm. **(G)** Representative Western blotting (top) and densitometric analysis (bottom) of GAP43 expression in diaphragms from 24-h-old mice, with brain extracts (Brain) used as the positive control for GAP43 expression. **(H–K)** Relative size frequencies of myofibrils **(H,J)** and fibers **(I,K)** in cross-sections of EDL muscle from 21-day-old **(H,I)** and 45-day-old **(J,K)** mice. p: Chi-square tests for trends; myofibrils in cross-sections of EDL from 21-day-old mice, n: 1515 WT, 1421 (+/−) and 1050 (−/−); fibers in cross-sections of EDL from 21-day-old mice, n: 385 WT, 400 (+/−) and 167 (−/−); myofibrils in cross-sections of EDL from 45-day-old mice, n: 406 WT, 406 (+/−) and 486 (−/−); fibers in cross-sections of EDL from 45-day-old mice, n: 197 WT, 81 (+/−) and 276 (−/−). **(L)** Percentages of transverse, oblique and longitudinal triads in longitudinal sections of EDL fibers from 21-day-old mice. **(M)** Percentage of triads localized in the intermyofibrillar space in longitudinal sections of EDL fibers from 21-day-old mice. Data are means ± SEM **(E,F,L,M)**. ^*^p < 0.05; ^**^p < 0.01; ^***^p < 0.001 (Students' *t*-tests).

To investigate the effects of the absence of GAP43 expression (i.e., GAP43^−/−^; GAP43 KO) in skeletal muscle, a morphometric analysis was performed on some of the muscles isolated from the GAP43^−/−^ mice. In fixed and isolated neonatal diaphragms from WT, GAP43^+/−^, and GAP43^−/−^ mice, the cross-sectional areas of the myofibrils and fibers were measured using electron microscopy. The relative frequencies of the myofibril and fiber cross-sectional areas were smaller for the fibers of the diaphragms of the neonatal GAP43^−/−^ mice, compared to those of the WT and GAP43^+/−^ mice (Figures 2A,B, Figure S1).

To determine the degree of diaphragm development, the orientations of the T-tubule and sarcoplasmic reticulum triads were evaluated in longitudinal sections for the WT, GAP43^+/−^, and GAP43^−/−^ neonatal diaphragms. The quantitative analysis of the orientation of the triads revealed that the diaphragms from the GAP43^−/−^ mice had significantly lower percentages of transverse triads, with respect to those from the WT and GAP43^+/−^ mice (Figure 2E, Figure S2). Another parameter that indicates the degree of muscle development is the fraction of T-tubules that are engaged in junction formation with the sarcoplasmic reticulum (Franzini-Armstrong, 1991). The extremely ordered organization of the functional ultrastructure of skeletal muscle fibers allows evaluation of the fraction of T-tubules that are engaged in junction formation, as indirectly determined according to the presence of triads in intermyofibrillar spaces in longitudinal sections. In these samples, when a triad is present in the intermyofibrillar space, there is a high probability that there will be engagement of T-tubules in junction formation. This parameter is expressed as the percentage of triads in the intermyofibrillar spaces for the total intermyofibrillar spaces analyzed. Therefore, a higher percentage of triads in the intermyofibrillar spaces reflects a higher percentage of T-tubules engaged in junction formation. The ultrastructural analysis of the neonatal diaphragm fibers from these GAP43^−/−^ mice revealed lower levels of triads per intermyofibrillar space, and thus lower percentages of T-tubules engaged in junction formation with the sarcoplasmic reticulum, as compared to the WT mice (Figure 2F).

Smaller cross-sectional areas of the myofibrils and fibers were also seen for adult (45-day-old) mouse diaphragms for the GAP43^−/−^ mice, in comparison to the WT mice (Figures 2C,D). However, these GAP43^−/−^ fibers showed the same triad orientations and percentages of triads in the intermyofibrillar spaces in comparison to the WT adult fibers (data not shown). Moreover, the analysis of GAP43 expression in the neonatal diaphragms showed that the GAP43^+/−^ mice had lower expression levels of GAP43, with respect to the WT mice (Figure 2G).

These analyses were also performed for the EDL muscles of two GAP43^−/−^ mice that survived to 21 and 45 days, ages at which the skeletal muscle fibers in the WT mice can be considered to be almost or completely mature, respectively (Takekura et al., 2001; Boncompagni et al., 2009). The GAP43^−/−^ 21-day-old EDL showed smaller diameters of the myofibrils and fibers than those for the WT and GAP43^+/−^ EDL (Figures 2H,I, Figure S1). Of note, the diameters of the myofibrils from the 45-day-old mice were similar to those in GAP43^+/−^ EDL mice, although the EDL from the 45-day-old GAP43^−/−^ mice had smaller fibers than for the WT and GAP43^+/−^ mice (Figures 2J,K).

In the same specimens, the analysis of triad orientation and localization in the EDL muscles for the 21-day-old mice revealed fewer transverse triads in the GAP43^−/−^ mice, in comparison with the WT and GAP43^+/−^ mice, and also showed lower percentages of T-tubules involved in junction formation with the sarcoplasmic reticulum (Figures 2L,M). The same analyses was performed in the EDL muscle from the 45-day-old GAP43^−/−^ mice, but did not show any differences in triad orientation and localization compared with the EDL muscles for the 45-day-old WT and GAP43^+/−^ mice (data not shown).

### Expression of GAP43 in skeletal muscle during development and neuromuscular junction formation

**Figure 3:**
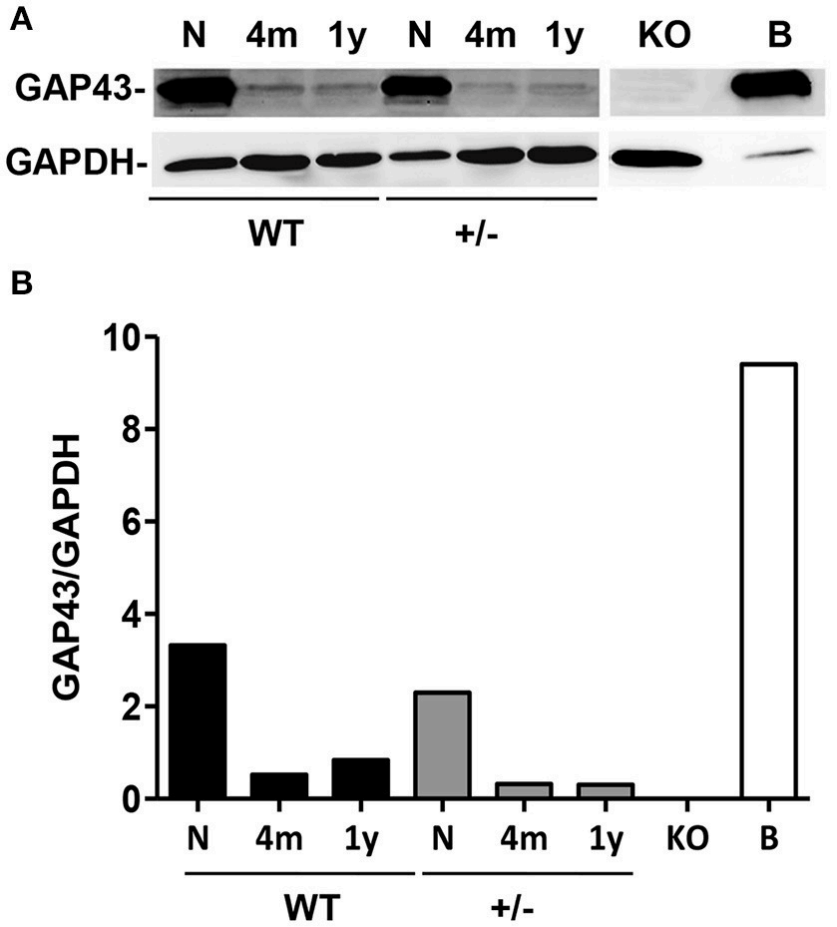
**Muscle GAP43 levels according to age. (A)** Representative Western blotting showing the bands that correspond to GAP43 in samples from muscles from the hind legs of newborn (N, 24 h after birth), adult (4 m, 4 months old) and old adult (1 y, 1 year old) WT and GAP43^+/−^ mice. KO, negative control from newborn limb muscles of GAP43^−^^/^^−^ mouse. **(B)** Positive control from brain extract of newborn WT mouse. **(B)** The corresponding densitometric analysis of the Western blotting in **(A)**.

The GAP43 expression levels were also determined during the development of the skeletal muscle in both WT and GAP43^+/−^ mice of different ages. Western blotting showed higher levels of GAP43 in the muscles of the hind legs of the newborn mice (i.e., 24 h old), when compared to these muscles of the adult (i.e., 4 months old) and older (i.e., 1 year old) WT and GAP43^+/−^ mice (Figure 3).

**Figure 4:**
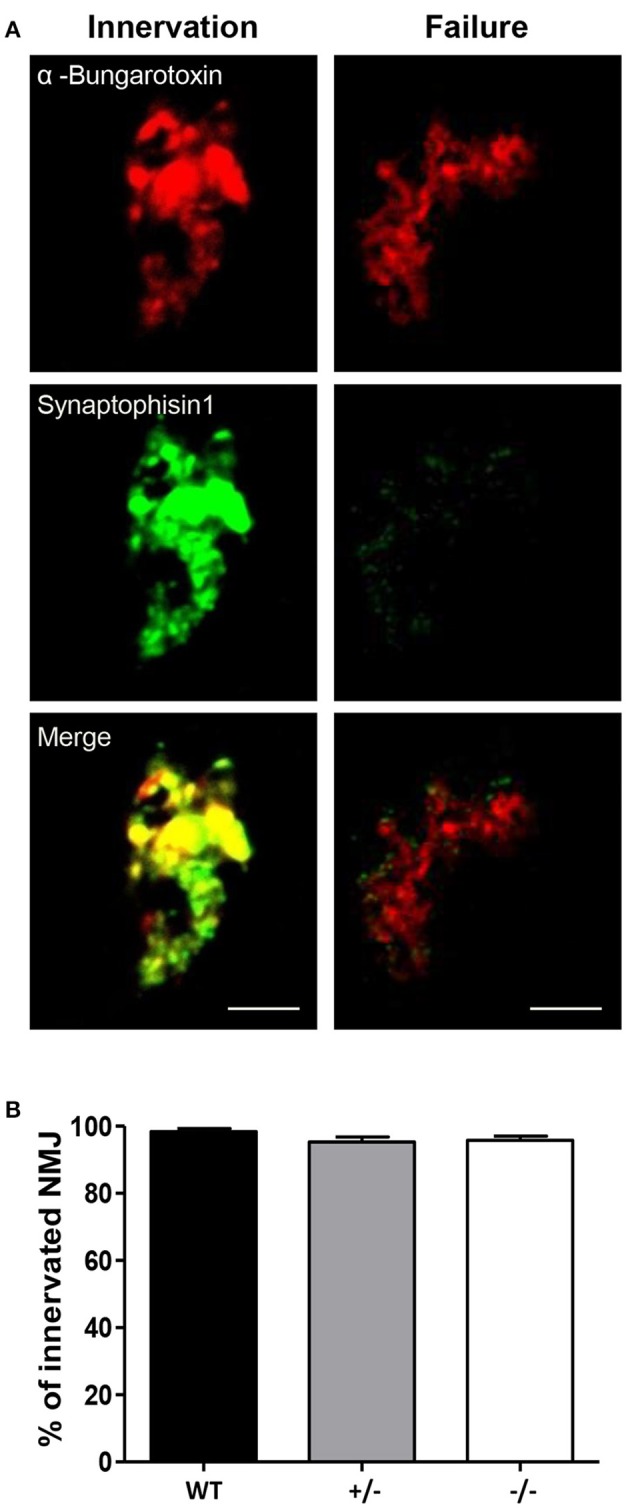
**Muscle innervations in the diaphragm. (A)** Representative images of the staining with rhodamine-labeled α-bungarotoxin (red) and an Alexa-conjugated anti-synaptophysin antibody (green), to show innervated and noninnervated (Failure) fibers. This staining was used to quantify the neuromuscular junction formation: the presence and absence of the yellow signal in the merge is indicative of innervation and noninnervation (Failure) of the fibers, respectively. Bar: 2 μm. **(B)** Percentages of innervated neuromuscular junctions (NMJ) for diaphragms from the WT, GAP43^+/−^ (+/−), and GAP43^−/−^ (−/−) mice. Data are means ± SEM (*n* = 3).

To determine whether the absence of GAP43 disrupts neuromuscular junction formation, the acetylcholine receptors on the newborn mouse diaphragms were labeled with rhodamine-labeled α-bungarotoxin, and synaptophysin (a pre-synaptic marker), and with an Alexa-conjugated anti-synaptophysin antibody (see representative images in Figure 4A). The α-bungarotoxin labeling revealed clusters of acetylcholine receptors, and in innervated fibers, this signal was coupled to the fluorescence of the antibody against the pre-synaptic marker synaptophysin. Here, innervation failure of fibers was revealed by the lack of the fluorescence signal of synaptophysin (Figure 4A). A large percentage of the neuromuscular junctions analyzed in the newborn mice diaphragms appeared to be innervated, and there were no differences in the diaphragms from GAP43^−/−^ mice, in comparison with the WT and GAP43^+/−^ mice (Figure 4B).

### Differentiation processes of GAP43^−/−^ satellite cells

**Figure 5:**
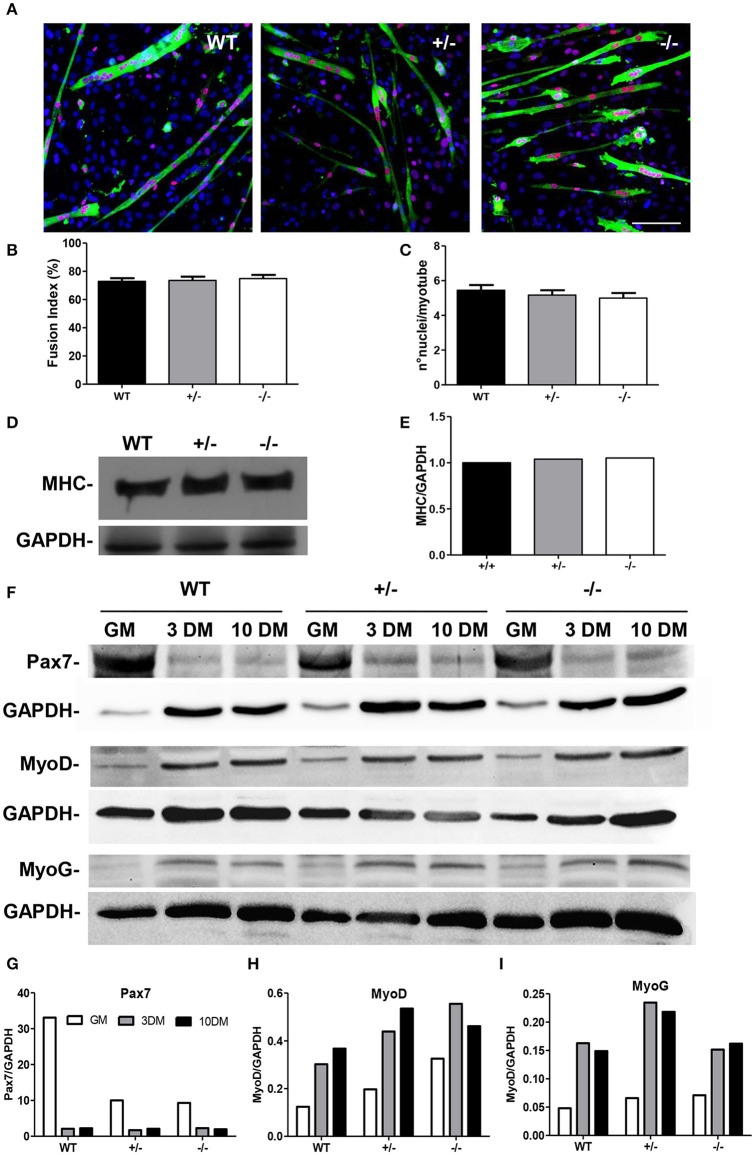
**Differentiation markers in satellite cells from the WT, GAP43^+/−^ (+/−), and GAP43^−/−^ (−/−) mice. (A)** Representative images of cells from murine muscles immuno-stained for MyoD (red) and MF20 (green), and for ToPro staining of the nuclei (blue). Bar: 100 μm. **(B)** Fusion Index (see text). **(C)** Myonuclear accretion (i.e., number of nuclei per myotube). Data are means ± SEM. **(D)** Representative Western blotting showing expression levels of myosin heavy chain (MHC) in cells from the WT, GAP43^+/−^ (+/−), and GAP43^−/−^ (−/−) mice, at 10 days in differentiation medium. **(E)** Corresponding densitometric analysis of the Western blotting in **(D)**. **(F)** Representative Western blotting showing expression levels of Pax7, MyoD, and MyoG in proliferating cells (growth medium, GM) or differentiating cells (3, 10 days in differentiation medium; 3 DM, 10 DM, respectively) from the WT, GAP43^+/−^ (+/−), and GAP43^−/−^ (−/−) mice. **(G–I)** The corresponding densitometric analyses of the Western blotting in **(F)**.

Satellite cells were isolated from the hind-limb muscles of the WT, GAP43^+/−^, and GAP43^−/−^ newborn mice, to study the effects of the lack of GAP43 expression on the myogenic program. The Fusion Index was calculated at 3 days of differentiation, a critical period during differentiation when the cells are triggered to differentiate into myotubes, although at this stage the process is not complete. The Fusion Index was quantified as the percentage of MyoD-positive nuclei located within multinucleated (≥2 nuclei) cells positive to sarcomeric myosin, compared to the total MyoD-positive nuclei (Figures 5A,B). A myonuclear accretion parameter was also evaluated, which is expressed as the mean number of nuclei per myotube (Figures 5A,C). These morphological analyses indicated that *in-vitro* the satellite cells from GAP43^−/−^ mice differentiated into myotubes to the same extent and with the same timing as for the WT and GAP43^+/−^ satellite cells. Indeed, the three phenotypes did not show significant differences. No significant differences were observed either for the analysis of the expression of specific markers during the *in-vitro* progression through the myogenic program. In particular, the expression levels of Pax7, MyoD, and myogenin (MyoG) were evaluated. These markers were analyzed in protein extracts from undifferentiated proliferating satellite cells that were isolated from the WT, GAP43^+/−^, and GAP43^−/−^ mice, and in protein extracts from the corresponding myotubes at 3 days and 10 days of differentiation (Figure 5F). The Western blotting revealed that the satellite cells and myotubes that differentiated *in-vitro* from the GAP43^−/−^ mice expressed all of the markers analyzed, and the expression levels and time courses of these proteins did not show any significant differences in comparison to those for the satellite cells from the WT and GAP43^+/−^ mice (Figures 5G–I). In addition, as a marker of the late myogenic differentiation, MHC also showed similar expression levels in extracts from myotubes at 10 days of differentiation (Figures 5D,E).

### Intracellular Ca^2+^ in the myotubes from GAP43^−/−^ mice

The intracellular Ca^2+^ levels were monitored in the differentiated myotubes derived from the WT, GAP43^+/−^, and GAP43^−/−^ mice (i.e., WT, GAP43^+/−^, and GAP43^−/−^ myotubes), using fluorescence video-imaging techniques and the fluorescent Ca^2+^ indicator, Fluo-4. The activation of the DHPR Ca^2+^ channel, the voltage-sensor of the CRUs, was tested by triggering membrane depolarization by adding 50 mM KCl to the medium. The activation of the RyR, as the inner component of the CRUs, was tested by adding 20 mM caffeine to the medium; as a RyR agonist, this concentration of caffeine induces Ca^2+^ release from the sarcoplasmic reticulum (Dettbarn et al., 1994; Herrmann-Frank et al., 1999).

**Figure 6:**
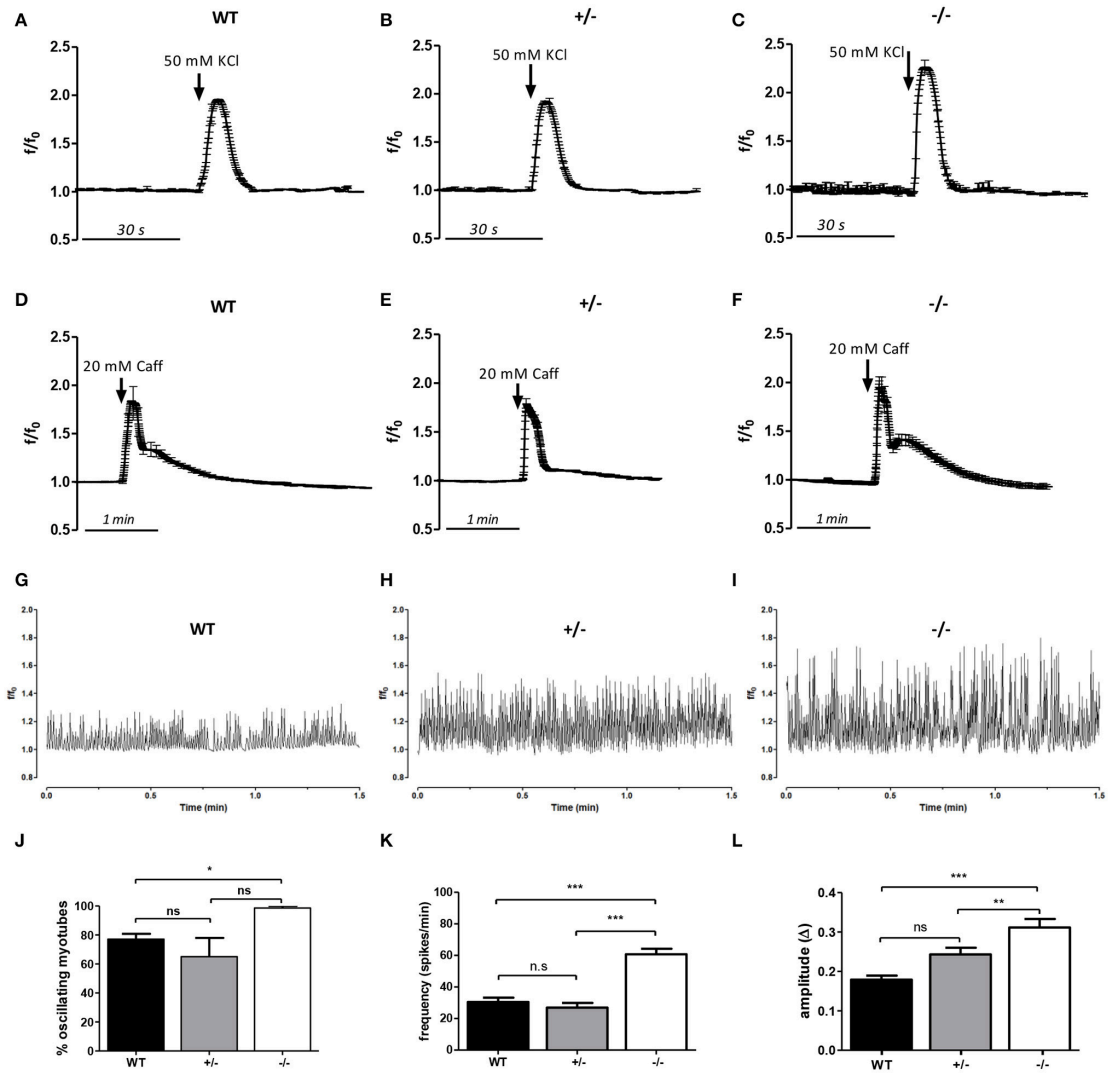
**Changes in intracellular Ca^2+^ levels in response to depolarization and caffeine stimulation, and during spontaneous cell activity in myotubes derived from the WT, GAP43^+/−^ (+/−), and GAP43^−/−^ (−/−) mice. (A–F)** Intracellular Ca^2+^ levels in response to 50 mM KCL and 20 mM caffeine (Caff). **(G–I)** Representative traces of intracellular Ca^2+^ oscillations. **(J–L)** Quantitative analyses of the spontaneous variations of the intracellular Ca^2+^ levels, as percentages of oscillating cells for each phenotype **(J)**, frequency of intracellular Ca^2+^ waves **(K)**, and amplitude of the waves **(L)**. Data are means ± SEM **(A–F,J–L)**. ^*^p < 0.05; ^**^p < 0.01; ^***^p < 0.001 (Students' *t*-tests).

The WT, GAP43^+/−^, and GAP43^−/−^ myotubes responded to KCl-induced depolarization and caffeine-stimulated RyR with intracellular Ca^2+^ increases (Figures 6A–F). The analysis of the kinetics of these Ca^2+^ increases showed some differences in the GAP43^−/−^ myotubes, when compared to the WT and GAP43^+/−^ myotubes. In particular, the quantitative analysis showed that in response to KCl, the GAP43^−/−^ myotubes had significantly higher peak Ca^2+^ amplitudes, and significantly faster intracellular Ca^2+^ increases in response to KCl (Figures 6A–C, Figure S3). Similar behavior was observed in response to caffeine: the Ca^2+^ release evoked by caffeine in the absence of extracellular Ca^2+^ in the GAP43^−/−^ myotubes had significantly greater amplitudes and faster intracellular Ca^2+^ increases when compared to the WT and GAP43^+/−^ myotubes (Figures 6D–F, Figure S3).

The intracellular Ca^2+^ dynamics in skeletal muscle are not only the primary mediator of muscle contraction, but are also involved in the regulation of many cellular activities that specifically require different Ca^2+^ concentrations according to amplitude and timing (Berridge et al., 1998; Dolmetsch et al., 1998; Berchtold et al., 2000). For this reason, the spontaneous cell activity was also monitored for the myotubes, by measuring the changes in the intracellular Ca^2+^. Also, the presence and analysis of spontaneous Ca^2+^ oscillations highlighted differences in the GAP43^−/−^ myotubes compared to the spontaneous activity present in the WT and GAP43^+/−^ myotubes. Ca^2+^ oscillations were seen for most of the myotubes analyzed, although the percentage of myotubes with oscillations was significantly higher in the GAP43^−/−^ myotubes with respect to the WT myotubes (Figures 6G–J). In addition, the analysis of the kinetics of these spontaneous Ca^2+^ oscillations revealed that the GAP43^−/−^ myotubes showed significantly higher frequency and amplitude of the Ca^2+^ oscillations, compared to the WT and GAP43^+/−^ myotubes (Figures 6K,L).

### L-type Ca^2+^ currents in the myotubes derived from the GAP43^−/−^ mice

**Figure 7:**
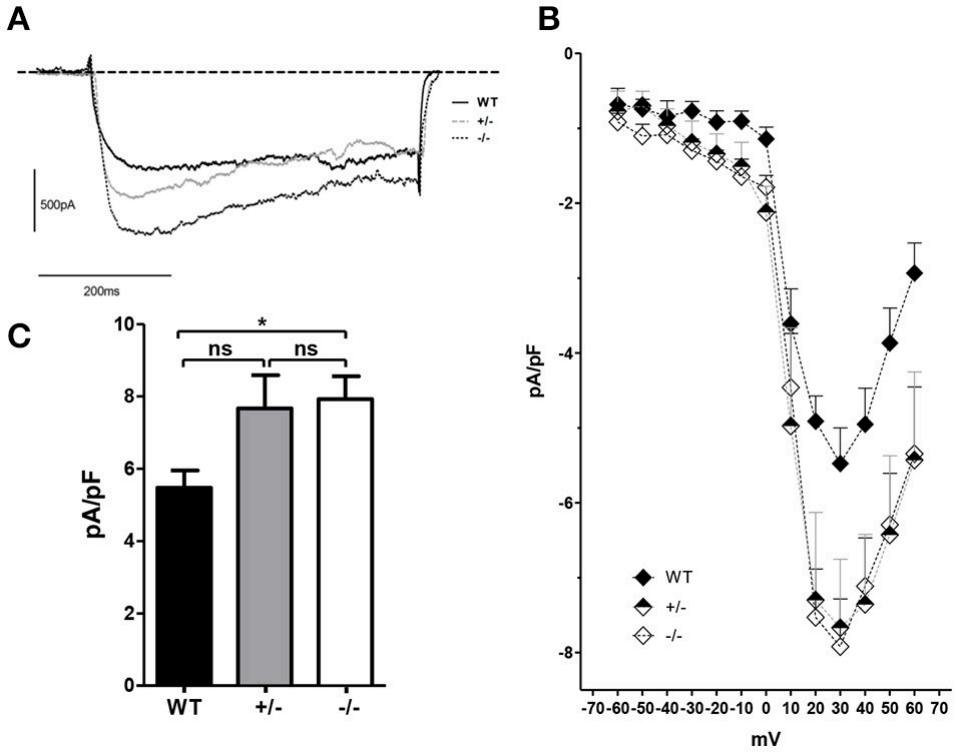
**L-type Ca^2+^ current recordings and analyses of myotubes derived from the WT, GAP43^+/−^ (+/−), and GAP43^−/−^ (−/−) mice. (A)** Representative traces of L-type Ca^2+^ currents recorded at +30 mV. **(B)** Current-voltage relationships for L-type Ca^2+^ currents. Data are means ± SEM of pA/pF, where for each cell pA is the current recorded at the test voltage with the respective cell capacitance (pF). **(C)** Current peaks normalized to the membrane capacitance (pA/pF) recorded at +30 mV. Data are means ± SEM. ^*^p < 0.05 (Students' *t*-tests).

The L-type Ca^2+^currents were analyzed in the WT, GAP43^+/−^, and GAP43^−/−^ myotubes (Figure 7). The experimental protocol was designed whereby the ramp stimulation was preceded by pre-stimulation at −30 mV that lasted 750 ms to reduce the contribution of the T-type Ca^2+^ currents. The L-type currents recorded at +30 mV appeared more pronounced in the GAP43^−/−^ myotubes, compared to the WT myotubes (Figure 7A). This was confirmed by the current/voltage ratios normalized to the membrane capacitance and the derived peak currents, which showed higher current intensities in the GAP43^−/−^ myotubes. These reached mean peak currents of approximately 8 pA/pF, compared to the lower mean peak currents in the WT myotubes, as approximately 6 pA/pF (Figures 7B,C). The L-type current recordings and analyses revealed heterogeneous behavior for the GAP43^+/−^ myotubes, which showed higher, although not significantly so, L-type currents with respect to those for the WT myotubes (Figure 7).

### GAP43 and CaM cross-talk

**Figure 8:**
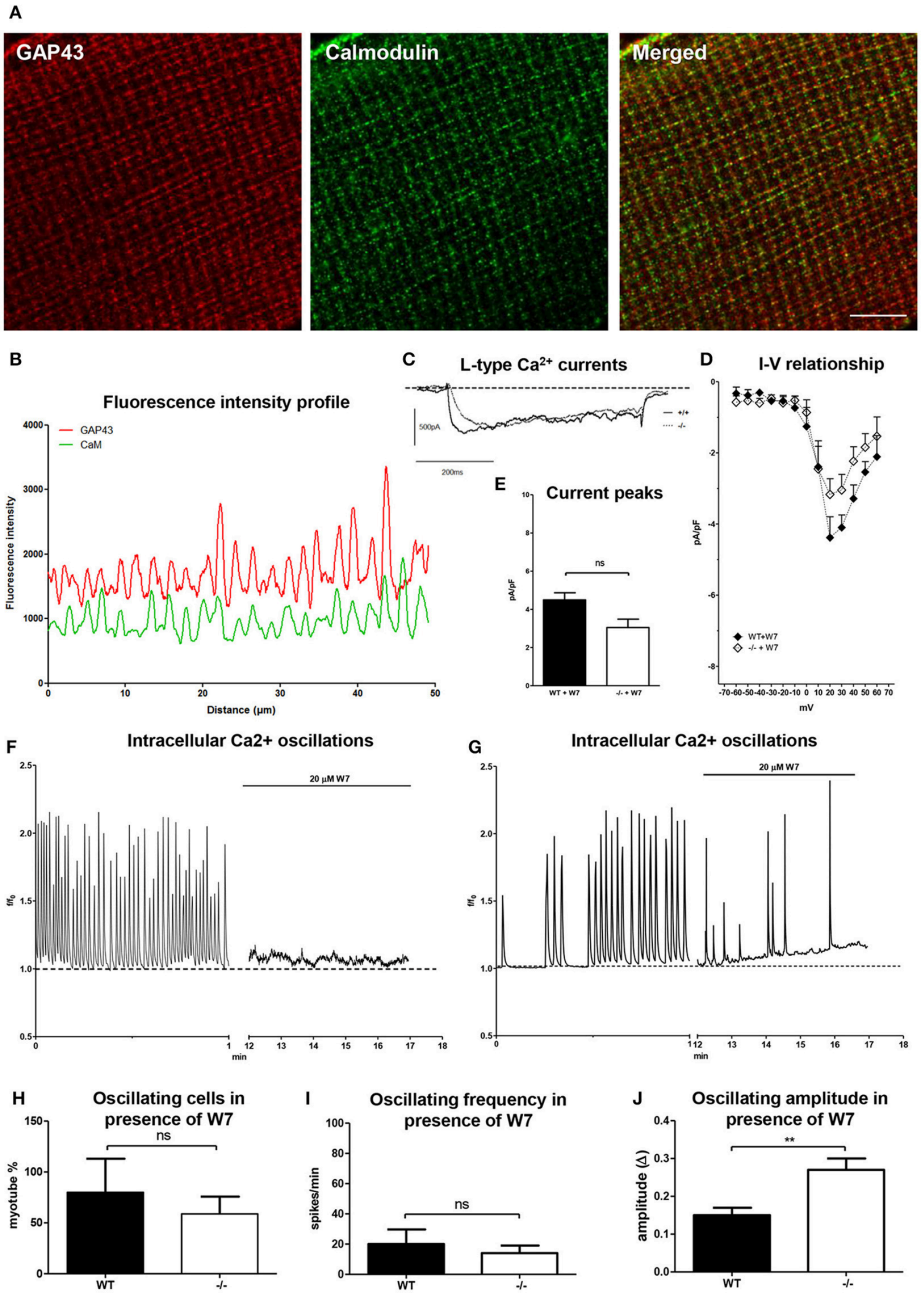
**GAP43/CaM localization in EDL muscle fibers and CaM involvement in intracellular Ca^2+^ handling in myotubes derived from the WT and GAP43^−/−^ (−/−) mice. (A)** Representative images of EDL muscle fibers double-stained with anti-GAP43 (red) and anti-CaM (green) antibodies. Bar: 10 μm. **(B)** Fluorescence intensity profiles as calculated from images as illustrated in **(A)**. **(C)** Representative traces of L-type Ca^2+^ currents recorded at +30 mV in myotubes in the presence of 20 μM W7. **(D)** Current-voltage relationships for L-type Ca^2+^ currents in myotubes in the presence of 20 μM W7. **(E)** Current peaks normalized to the membrane capacitance (pA/pF) recorded at +30 mV in myotubes in the presence of 20 μM W7. **(F,G)** Representative traces for intracellular Ca^2+^ in myotubes from both WT and GAP43^−/−^ (−/−) mice. **(H–J)** Quantitative analyses of spontaneous intracellular Ca^2+^ oscillations in myotubes after addition of 20 μM W7, as the percentage of cells with Ca^2+^ oscillations **(H)**, the frequency of the Ca^2+^ oscillations **(I)**, and the amplitude of the Ca^2+^ oscillations **(J)**. Data are means ± SEM **(D,E,H,I,J)**. ^**^p < 0.01 (Students' *t*-tests).

To investigate the relationships between GAP43 and CaM in the handling of the intracellular Ca^2+^ levels in these skeletal muscle phenotypes, the relative localization between GAP43 and CaM was monitored, and a pharmachological approach was also used. Double immunostaining for GAP43 and CaM was performed for the EDL muscle fibers derived from 3-month-old WT adult mice. The images obtained showed regular patterns of spotted transverse striations for both GAP43 and CaM, which were in close proximity to each other (Figure 8A). The localization of GAP43 and CaM was also illustrated by the computed fluorescence intensity profile along a line that depicts the GAP43 through a single myofiber in a fluorescence image (Figure 8B). Along the fibers, the maxima of the intensities of the specific fluorescence for GAP43 and CaM showed high levels of red/green overlap (Figure 8B).

This close proximity of GAP43 and CaM leads to the possibility of cross-talk between these two proteins, with potentially mutual influences on intracellular signaling. For this reason, the L-type Ca^2+^ currents and spontaneous intracellular Ca^2+^ waves were investigated further in these cell models in the presence of the selective CaM-blocking agent, W7 (N-[6-aminohexenyl]-5-chloro-naphthalenesulfonamide). After a pre-incubation for 10 min with 20 μM W7 (which was present in the medium also during the recordings) the L-type Ca^2+^ currents were recorded. These appeared to be reduced in the GAP43^−/−^ myotubes, in comparison to the absence of W7 (see Figure 7A). With the addition of the W7 CaM blocker, there were no significant differences between the L-type Ca^2+^ currents at +30 mV for the WT and GAP43^−/−^ myotubes (Figure 8C). The profile of the L-type Ca^2+^ current/voltage ratios did not show any shift in the channel kinetics (Figure 8D). Quantitative analyses of the L-type Ca^2+^ currents normalized to the cell capacitance (Figure 8E) showed that with the addition of W7 there was marked reduction (up to about 50%) in the L-type Ca^2+^ current amplitude and peak in the GAP43^−/−^ myotubes, while there were no significant changes in these parameters for the L-type Ca^2+^ current in the WT myotubes (compare Figure 8E and Figure 7C).

In the presence of W7, the spontaneous Ca^2+^ oscillations were also modified. These spontaneous Ca^2+^ oscillations stopped in approximately half of the GAP43^−/−^ myotubes, and in about 20% of the WT myotubes, compared to the absence of W7 (compare Figures 8F,G to Figure 6). Quantitative analysis of the kinetics of the intracellular Ca^2+^ variations in the remaining active myotubes (Figure 8H) revealed lower frequencies (Figure 8I), but unaltered amplitudes (Figure 8J) of these Ca^2+^ oscillations, in comparison to those in the absence of W7 (Figure 6).

## Discussion

In this study, we investigated the role of GAP43 in skeletal muscle tissue initially by highlighting some of the macroscopic morphometric and functional parameters of the GAP43^−/−^ mice. Then, we focused our attention on the ultrastructural analysis of the skeletal muscle of the GAP43^−/−^ mice, and investigated the intracellular Ca^2+^ dynamics in the myotubes derived from satellite cells of these mice.

As also reported in other studies (Strittmatter et al., 1995; Maier et al., 1999; Metz and Schwab, 2004), in the GAP43^−/−^ mice in the present study, GAP43 expression was not required for embryonic viability, although a high percentage of the GAP43^−/−^ mice do not survive beyond weaning. This indicates a role for GAP43 in post-natal development, and above all in those tissues in which particular plasticity processes are present, such as the nervous system and skeletal muscle (Rice and Barone, 2000). The GAP43^−/−^ newborn mice had similar body weights to those of the WT mice, but the adult GAP43^−/−^ mice showed significantly lowered body weight and altered posture and motor control. It can be speculated that the smaller body weight is the result of the inability of the GAP43^−/−^ mice to adequately feed, even though the GAP43^−/−^ mice that survived had the same access to breast feeding with the mother and to food and water, in comparison to the WT mice. The lower body weight might mean lower skeletal muscle mass, and hence justify the lower grip-test performance, and indirectly muscle strength, shown by the GAP43^−/−^ mice in comparison to the WT mice.

The levels of GAP43 expression are critical not only for nervous system development and growth (Jain et al., 1995; Murata et al., 2005), but also for skeletal muscle development after birth. Indeed, during aging the GAP43^+/−^ mice also showed decreased body weight and muscle strength in comparison to the WT mice. Furthermore, as other studies have also reported for specific areas of neuronal tissue (Jain et al., 1995; Murata et al., 2005), in the WT mice, from birth to old age, the GAP43 expression decreased when muscle maturation was complete (i.e., at 1 to 4 months old) and remained low during old age (i.e., in 1 year old). In addition, the expression of GAP43 was a little lower in the muscle derived from the heterozygous GAP43^+/−^ mice, in comparison to the WT mice. These data suggest that the levels of GAP43 at birth are a critical factor for ongoing development.

To check this hypothesis, we monitored the ultrastructural organization of the skeletal muscle from newborn mice and from mice at 45 days of age, when the skeletal muscle can be considered to be fully developed (Boncompagni et al., 2009). The homogeneous and regular architecture of the adult skeletal muscle fibers defined the close relationship between their structure and function, and the use of electron microscopy revealed the degree of development of the skeletal muscle fibers (Stromer et al., 1974; Romero et al., 2013). We performed morphometric analyses on diaphragm muscle fibers from the newborn WT and GAP43^−/−^ mice. The choice of diaphragm was because this muscle has an almost mature structure at birth (Franzini-Armstrong, 1991), and allows the study of the muscle fiber architecture and the localization of the functional structures, such as the triads and CRUs. These data showed that in comparison to diaphragms from newborn WT mice, those from newborn GAP43^−/−^ mice had smaller myofibrils and fibers. In addition, they had a delayed degree of maturation, as seen by the higher levels of longitudinal triads and less triad-junction formation. These last two features disappeared in the diaphragms of 45-day-old GAP43^−/−^ mice, an age at which the development of skeletal muscle can be considered complete (Boncompagni et al., 2009).

Similar behavior was observed also for the EDL muscle. Indeed, in comparison to the EDL muscle from the WT mice, that from the 21-day-old GAP43^−/−^ mice had higher levels of longitudinal triads and lower triad-junction formation. Again, however, the EDL muscle from 45-day-old GAP43^−/−^ mice did not show significant differences compared to that from the WT mice. Overall, this suggests that the lack of GAP43 expression does not prevent the development of skeletal muscle, but instead causes its delay. This is probably due to modifications to the muscle intracellular signaling pathways, as the degree of innervation of these GAP43^−/−^ mouse muscles was not altered with respect to that of the WT mouse muscles.

To determine whether the lack of GAP43 causes modifications to intracellular signaling, we used an *in-vitro* cell model of skeletal muscle differentiation: the satellite cells. These cells represent the adult stem cells of skeletal muscle tissue, so they have the same genotype, and they are responsible for the muscle regeneration processes. Indeed, they are “conditioned” by the extracellular muscle microenvironment from which they are isolated, and they also establish functionally mature myotubes *in-vitro* (Musarò and Barberi, 2010). The satellite cells derived from the GAP43^−/−^ newborn mice showed morphological characteristics, myogenic potential, and differentiation timing that were similar to the satellite cells derived from the WT mice. Even if these macroscopic biological features were not modified by the lack of GAP43 expression, some of the functional properties were analyzed in the myotubes differentiated from these isolated satellite cells, including intracellular Ca^2+^ variations and the electrical properties of the sarcolemma.

Ca^2+^ is fundamental for intracellular signaling, and more importantly, it is a key player in the functional activity of skeletal muscle, which primarily represents the contraction process and strength expression. As with the myotubes that were differentiated from the satellite cells from the WT mice, those isolated from the GAP43^−/−^ mice showed intracellular Ca^2+^ variations that were peculiar to the skeletal muscle phenotype, and included: spontaneous Ca^2+^ oscillations, voltage-operated channel activation, and RyR channel opening. Interestingly, the entity and kinetics of the intracellular Ca^2+^ dynamics, as well as the L-type Ca^2+^ currents, were different in the GAP43^−/−^ myotubes, in comparison to those observed in the WT myotubes.

Indeed, in the absence of GAP43 expression, the spontaneous and induced intracellular Ca^2+^ variations had greater amplitudes and were of higher frequency, which suggested dysregulation of Ca^2+^ homeostasis. For all of the experimental approaches used in the present study (from the *in-vitro* to the *in-vivo* analyses), the GAP43^+/−^ samples showed more heterogeneous behavior that was between that observed in the GAP43^−/−^ samples and that observed in the WT samples. Also, the amplitude and velocity of the intracellular Ca^2+^ oscillations recorded for the GAP43^−/−^ myotubes were intermediate to those recorded in the WT and the GAP43^−/−^ myotubes.

This suggests that the presence and expression levels of GAP43 can affect the intracellular Ca^2+^ dynamics, even if there is no evidence of any direct involvement of GAP43 in Ca^2+^ homeostasis. Indeed, it is known that the GAP43 structure contains an IQ domain, which is a binding site for the Ca^2+^-binding protein CaM. CaM acts as a common Ca^2+^ sensor for many signaling pathways, and it transduces local Ca^2+^ signals into specific cellular outcomes. The IQ domain of GAP43 mainly binds apoCaM (i.e., at low Ca^2+^ concentrations), and releases it when Ca^2+^ increases or when GAP43 is phosphorylated by PKC (Kumar et al., 2013). In neuronal tissues and cells, the GAP43/CaM interaction was shown to be involved mainly in membrane/cytoskeleton remodeling (Gamby et al., 1996; Denny, 2006).

In skeletal muscle, there are numerous functional proteins that contain IQ domains (e.g., L-type voltage-operated channels, DHPR, and RyR channels), and consequently their biological activities are modulated by CaM. In addition, CaM is present inside cells in at least two forms: a Ca^2+^-free form (ApoCaM), and a Ca^2+^-bound form (Ca^2+^-CaM), which depends on the intracellular free Ca^2+^ concentrations (Török et al., 1992). The activity of DHPR is Ca^2+^-dependent, and this modulation is mediated by CaM. In particular, the L-type Ca^2+^ channel has different CaM-binding domains, and its protein-protein interactions (and consequently its channel activity) depend on the Ca^2+^-induced conformational state of CaM, thus resulting in an important mechanism for fine tuning the regulation of intracellular Ca^2+^, and consequently of skeletal muscle function (Stroffekova, 2008, 2011). On the other hand, CaM also binds to the RyR channels of skeletal muscle. In particular, at low Ca^2+^ concentrations, ApoCaM can activate these channels to induce their opening, while Ca^2+^-bound CaM inhibits this (Yamaguchi et al., 2001; Samsó and Wagenknecht, 2002).

Considering all of this evidence and the altered Ca^2+^ handling in the myotubes from the GAP43^−/−^ mice, we investigated the relationships between GAP43 and CaM in our experimental models. This involved analysis of the intracellular localization of CaM in relation to the regular positioning of GAP43, and of the Ca^2+^ movements in the presence of the CaM inhibitor, W7, which is widely used for biochemical studies of Ca^2+^ -CaM-dependent signaling pathways. Among other CaM antagonists, W7 shows a selective action on CaM and it is not toxic to the cells at concentrations from 20 to 600 μM (Hidaka et al., 1981; Osawa et al., 1999; Suzuki et al., 1999). These data revealed that in the WT fibers, CaM localized in close proximity to GAP43. In particular, complete overlap was not seen for the tagged fluorescent signals that indicated the localization of CaM and GAP43, and thus this suggests that they can interact dynamically, and that CaM is also partially free in the cytoplasm. In the functional experiments, the presence of the CaM inhibitor restrained the effects due to the absence of GAP43 expression. Indeed in the GAP43^−/−^ myotubes, W7 decreased the intracellular Ca^2+^ oscillation frequency and the Ca^2+^ current amplitude. These data indicate that the effects due to the absence of GAP43 expression are mediated, at least in part, by the lack of a regulatory mechanism for CaM signaling.

The data obtained here provide the first evidence concerning the role of GAP43 in mouse skeletal muscle, both in this tissue *in-vivo* and in myotubes from the adult satellite cells cultured *in-vitro*. GAP43 expression appears to be involved in the timing of muscle maturation *in-vivo*. On the other hand, the absence of GAP43 does not affect the differentiation processes of satellite cells *in-vitro*.

Of note, the GAP43 involvement in Ca^2+^ handling is clear. We hypothesize that by interacting with CaM, GAP43 indirectly modulates the activity of DHPR and RyR channels, and also the spontaneous Ca^2+^ oscillations in myotubes. The location of GAP43 close to the CRUs in skeletal muscle generates a “functional microdomain” that anchors CaM near to the CRUs. These results in the fine tuning of the CaM regulatory activity for the DHPR and RyR channels, and consequently for intracellular Ca^2+^ handling, and thus muscle contraction. In addition, considering that GAP43/CAM binding is regulated by PKC and calcineurin, their roles will not be confined to the contraction process, but will extend over other cellular processes, such as enzymatic activity, cell metabolism, and gene expression (Bueno and Molkentin, 2002; Adiga and Nair, 2008; Wang et al., 2015).

The absence of GAP43 and the failure of GAP43/CaM binding would result in a higher level of free intracellular CaM. With this no longer buffered by GAP43, CaM might interact differently with the DHPR and RyR channels, thus altering their activities, and also modifying spontaneous Ca^2+^ movements.

In conclusion, these data now pave the way to an exciting hypothesis that indicates the key roles for the GAP43/CaM interaction in skeletal-muscle activity. In addition, these data open new perspectives in this research field, which thus require deeper investigation in the future.

## Author contributions

Conceived and designed the experiments: GC, CM, SG, MM. Performed the experiments: GC, CM, SP, SG. Analyzed the data: GC, CM, SP, RN, SG. Contributed to the writing of the manuscript: GC, SG, MM.

### Conflict of interest statement

The authors declare that the research was conducted in the absence of any commercial or financial relationships that could be construed as a potential conflict of interest.
