# Supplementary figures and images for: Evidence for Altered Ca2+ Handling in Growth Associated Protein 43-Knockout Skeletal Muscle
Source: Front Physiol. 2016 Oct 26;7:493. doi: 10.3389/fphys.2016.00493 (PMC5080375; doi:10.3389/fphys.2016.00493)

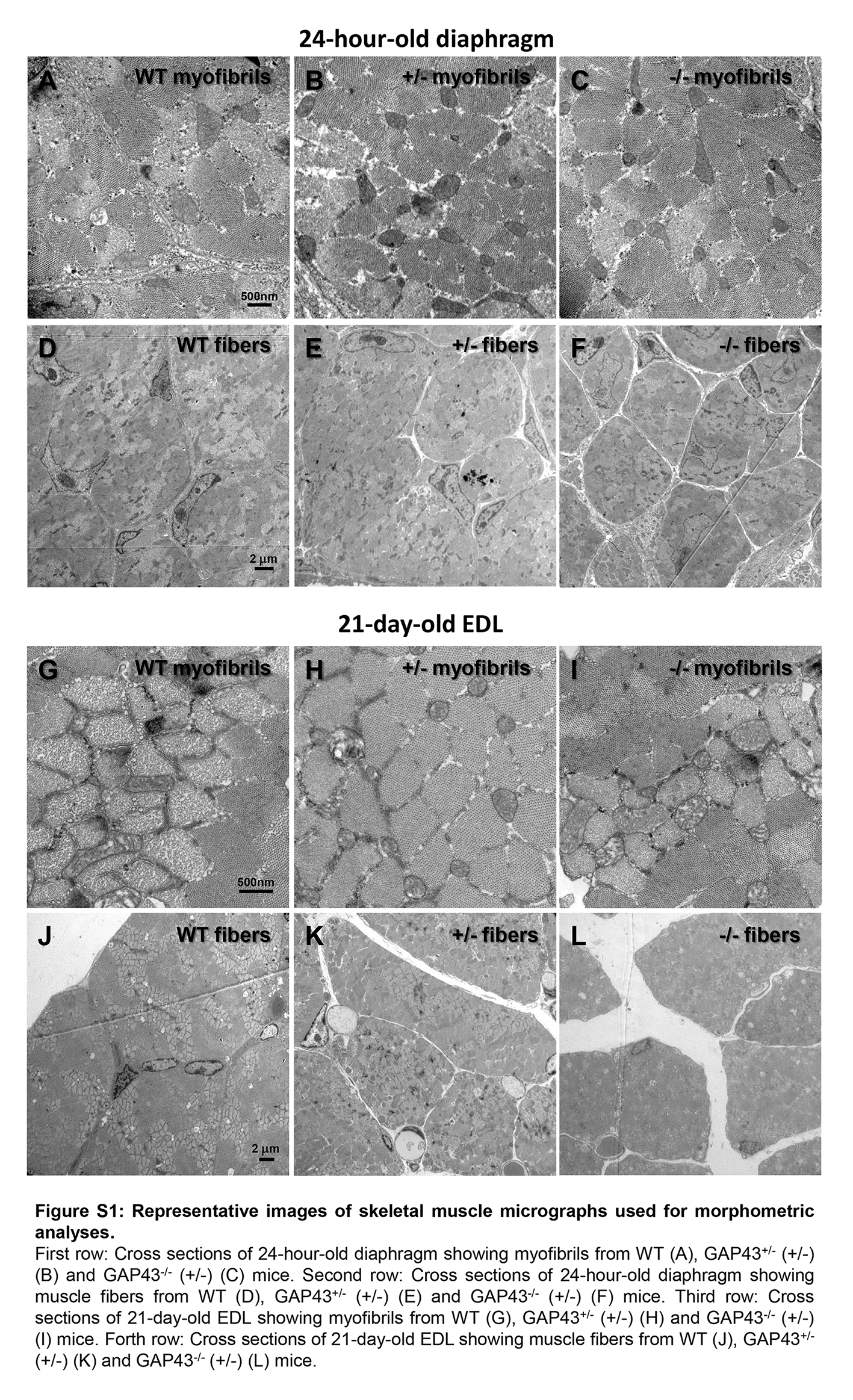

Supplement: Supplementary file 1 [file Image1.TIF]

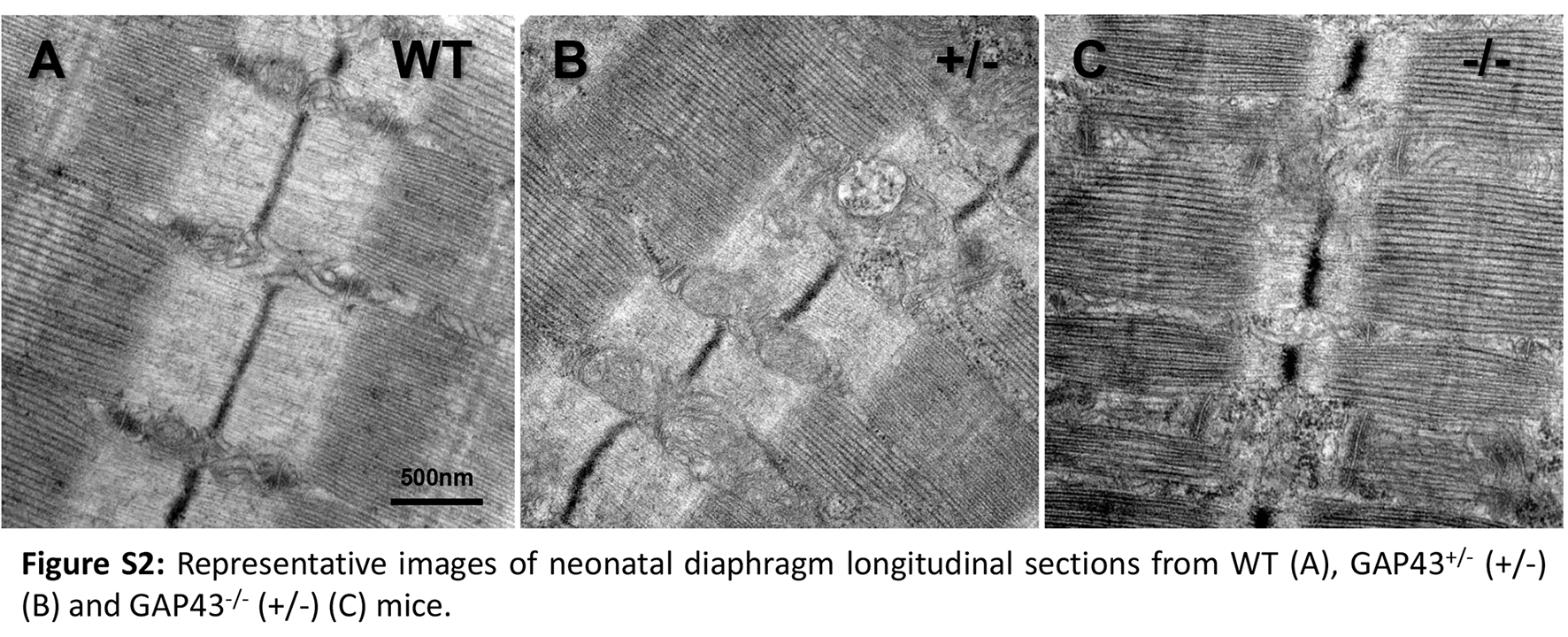

Supplement: Supplementary file 2 [file Image2.TIF]

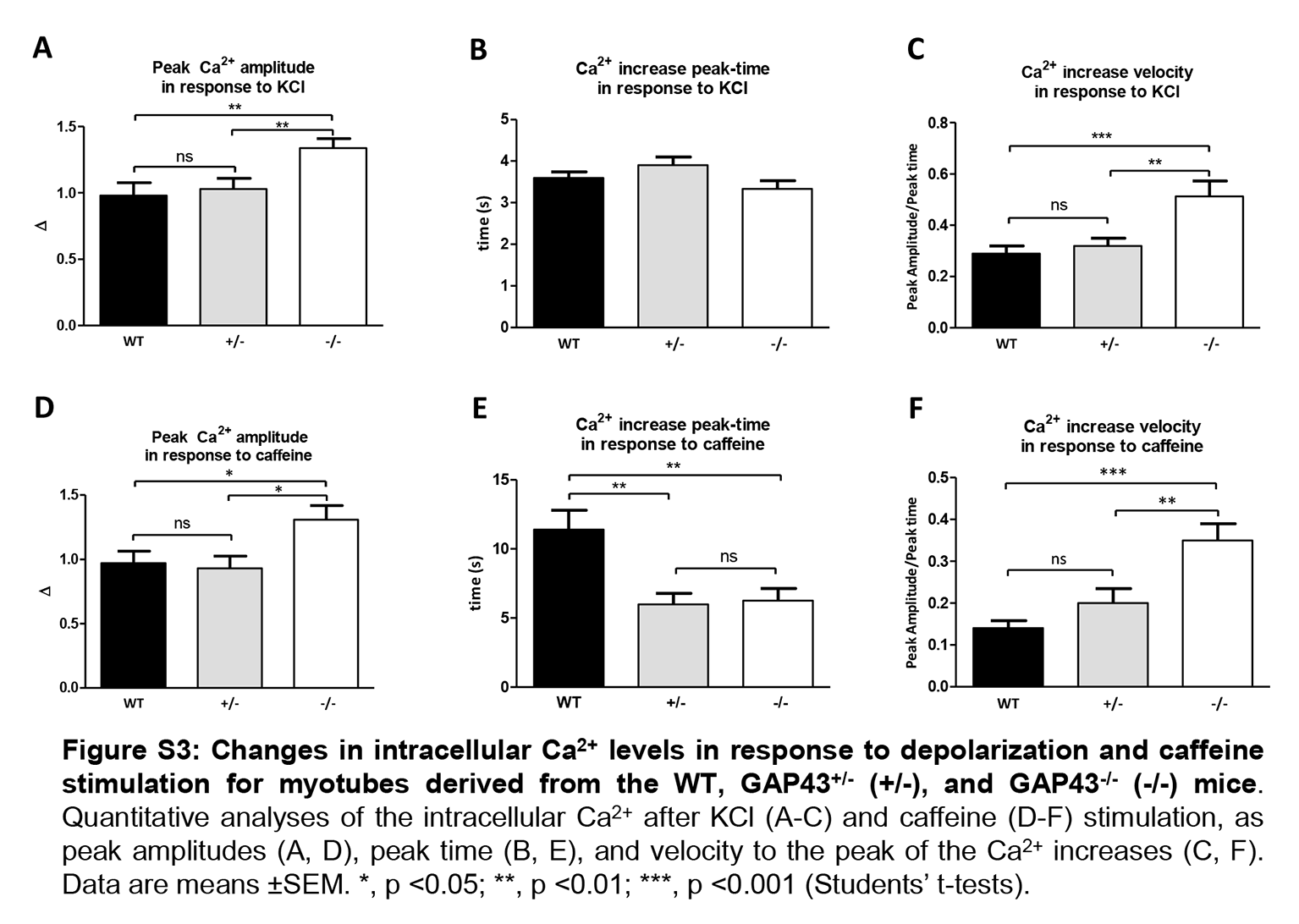

Supplement: Supplementary file 3 [file Image3.TIF]
